# Analgesic Effect of the Lysine-Containing Short Peptide Is Due to Modulation of the Na_V_1.8 Channel Activation Gating System

**DOI:** 10.3390/life13091800

**Published:** 2023-08-24

**Authors:** Arina D. Kalinina, Ilya V. Rogachevskii, Dmitriy M. Samosvat, Georgy G. Zegrya, Irina P. Butkevich, Viktor A. Mikhailenko, Vera B. Plakhova, Valentina A. Penniyaynen, Svetlana A. Podzorova, Boris V. Krylov

**Affiliations:** 1 Pavlov Institute of Physiology, Russian Academy of Sciences, 199034 Saint Petersburg, Russiaverapl@mail.ru (V.B.P.);; 2 Ioffe Institute, Russian Academy of Sciences, 194021 Saint Petersburg, Russia

**Keywords:** lysine-containing peptides, Na_V_1.8 channel, patch-clamp method, organotypic tissue culture method, formalin test, conformational analysis, nociception, analgesics

## Abstract

The present work continues our recent series of articles that aim to elucidate the ligand–receptor binding mechanism of short cationic peptides to the Na_V_1.8 channel in the nociceptive neuron. The applied methodological approach has involved several methods: the patch-clamp experimental evaluation of the effective charge of the Na_V_1.8 channel activation gating system, the organotypic tissue culture method, the formalin test, and theoretical conformational analysis. The lysine-containing short peptide Ac-KEKK-NH_2_ has been shown to effectively modulate the Na_V_1.8 channel activation gating system. As demonstrated by the organotypic tissue culture method, the studied short peptide does not trigger the downstream signaling cascades controlling neurite outgrowth and should not be expected to evoke adverse side effects. Conformational analysis of the Ac-KEKK-NH_2_ molecule has revealed that the distances between the positively charged amino groups of the lysine side chains are equal to 11–12 Å. According to the previously suggested mechanism of ligand–receptor binding of short peptides to the Na_V_1.8 channel molecule, Ac-KEKK-NH_2_ should exhibit an analgesic effect, which has been confirmed by the formalin test. The data obtained unequivocally indicate that the studied lysine-containing short peptide is a promising candidate for the role of a novel analgesic medicinal substance.

## 1. Introduction

Pain is essential for the survival and defense of the human organism. It is generally accepted that pain that becomes chronic is pathological [1]. The long-term systemic use of analgesics often evokes irreversible adverse side effects. These effects can be particularly severe upon administration of opioid drugs, which can be the only protocol drugs of choice for medicinal treatment of a wide variety of pathological states. Currently, the problem of the “opioid crisis” remains unresolved, the essence of which is that the uncontested need for the use of opioid drugs leads to overdose and death [2]. Quality of life indicators in patients suffering from chronic pain can only be corrected with adequate pain management using medicinal substances without adverse side effects and methods of psycho-emotional support [1,3,4].

To help solve the global problem of chronic pain, we have proposed a novel approach to the creation of safe and effective analgesics. It is based on the search for substances of an endogenous nature that can effectively modulate the functional activity of Na_V_1.8 channels. These channels are molecular markers of the nociceptive neurons [5]. According to our hypothesis, a decrease in their functional activity eliminates only the high-frequency component of the nociceptive neuron membrane impulse firing [6]. It is this component that transfers information about pain to the central nervous system. At the same time, the low-frequency components of impulse firing remain unaffected, which makes it possible to inform the brain about other adequate sensory signals generated by the polymodal nociceptors. Thus, medicinal substances specifically modulating the Na_V_1.8 channel functional activity can be qualified for the role of effective and safe analgesics without adverse side effects. 

Previously, we have designed and investigated some arginine-containing short peptides (Ac-RRR-NH_2_, H-RRR-OH, Ac-RERR-NH_2_, Ac-PRARRA-NH_2_, Ac-PRERRA-NH_2_) which significantly decrease the effective charge transferred by the Na_V_1.8 channel activation gating system (Z_eff_), being applied at 100 nM [7,8,9]. The observed decrease in this parameter due to the effects of the indicated peptides modulates the electrophysiological characteristics of the Na_V_1.8 channel in such a way that the channel becomes unable to generate the high-frequency impulse firing in the nociceptive neuron membrane. The complex methodological approach combining theoretical and experimental techniques (conformational analysis, the patch-clamp method, and the organotypic tissue culture method) has been implemented to elucidate the mechanism of ligand–receptor binding of arginine-containing short peptides to their suggested molecular target, the Na_V_1.8 channel [8,9]. 

The arginine side chain contains at its end the guanidinium group, which carries a positive charge even in the bulk of a protein [10] and can participate in the formation of intermolecular ionic bonds with the appropriate functional groups of the Na_V_1.8 channel molecule upon ligand–receptor binding of the peptides. Conformational analysis has made it possible to determine the distance between the guanidinium groups as the structural parameter that correlates with the observed experimental effect of a number of arginine-containing short peptides [9]. It should be stressed that all arginine-containing peptides studied by us earlier (3–6 amino acid residues) were designed and synthesized using only four different building blocks, arginine (R), glutamic acid (E), alanine (A), and proline (P), in order to not make the peptide molecules very structurally diverse. However, several peptides were not reported to modulate the Na_V_1.8 channel functional activity at all, which allowed us to estimate that the distances between the guanidinium groups should fall within the range of 9–12 Å to provide sufficient steric and electrostatic ligand–receptor complementarity. We suggest that the theoretical evaluation of this parameter should help design short peptides with a specific modulating effect on the Na_V_1.8 channel activation gating system. This approach not only opens up new principles of development of effective and safe, due to their endogenous nature, peptide analgesics but also helps elucidate the role of protein–protein interactions in the nociceptive system of the brain.

The next logical step in the computational protocol would be to dock the peptide with the Na_V_1.8 channel molecule. Unfortunately, it is not so straightforward specifically for this channel out of all other voltage-gated sodium channels. The experimental structure of the Na_V_1.8 channel has been obtained only very recently using the cryogenic electron microscopy technique [11]. It has been demonstrated that the voltage-sensing domain VSD_I_ in the Na_V_1.8 channel molecule is structurally different from those in the other members of the Na_V_1.x superfamily. However, the VSD_I_ domain conformation has not been well resolved, as opposed to the corresponding domains in the other voltage-gated sodium channels [12,13,14,15,16,17]. The authors consider that VSD_I_ represents a continuum of rather distinct conformations, which makes it difficult to draw reliable conclusions based on the docking of a single ligand to a single model of VSD_I,_ as the domain seems to be rather flexible specifically in the Na_V_1.8 channel. Therefore, to obtain detailed insight into the molecular mechanisms of ligand–receptor binding of the peptides to the Na_V_1.8 channel molecule, it is required to dock several peptide molecules with several VSD_I_ domain models, which is a challenging task beyond the scope of the current investigation. 

In the present study, we have attempted to substitute the arginines in a short peptide molecule for the lysines. Lysine (K) is the only naturally occurring amino acid, except for arginine (R), that contains a positively charged functional group at the end of a long and flexible side chain, which is the amino group. The lysine-containing peptide, Ac-KEKK-NH_2_, the analog of one of the active arginine-containing peptides, Ac-RERR-NH_2_, has been synthesized, and its effects were investigated in the framework of our methodological approach. Within this approach, the patch-clamp method is implemented to study the target physiological effect, the decrease in Z_eff_ due to extracellular application of a peptide molecule to the nociceptive neuron membrane; the organotypic tissue culture method helps investigate the possible neurite-inhibiting effect of a peptide, which could result in adverse side effects upon its medicinal use; conformational analysis is used to correlate the structural parameters of peptide molecules with their observed experimental effects. The results reported below for Ac-KEKK-NH_2_ strongly support our idea that ligand–receptor binding of the lysine- and arginine-containing short peptides is controlled by intermolecular bonds of a predominantly ionic nature. The analgesic effect of the peptide has been observed in vivo in behavioral experiments.

## 2. Materials and Methods

### 2.1. Chemicals and Reagents

All chemicals, excluding Ac-KEKK-NH_2_, were purchased from Sigma (Sigma-Aldrich, St. Louis, MA, USA). Ac-KEKK-NH_2_ was synthesized in the Verta Research and Production Company (St. Petersburg, Russia) by the method of classic peptide synthesis using reagents from Sigma (Sigma-Aldrich, St. Louis, MA, USA) and Iris Biotech GmbH (Marktredwitz, Germany) and characterized with high-performance liquid chromatography (purity of more than 95%) and mass spectrometry (see Supplementary Materials).

### 2.2. Patch-Clamp Method

Dissociated sensory neurons were obtained from the dorsal root ganglia (DRG) of newborn Wistar rats in accordance with the protocol described in much detail earlier, which allows us to record the Na_V_1.8 currents only [7,8,9,18,19,20]. 

Ligand–receptor binding of the lysine-containing short peptide Ac-KEKK-NH_2_ to the Na_V_1.8 channel was studied at the same concentration, 100 nM, as used earlier for the arginine-containing peptides [8,9]. This channel belongs to the sodium channel superfamily (Na_V_1.1–Na_V_1.9) [21,22]. Remarkably, the Na_V_1.8 channel inactivation gating system is characterized by slow relaxation, which makes it possible to separate in time the processes of activation and inactivation when the channel opens in response to a depolarizing step of the transmembrane potential E. Another sodium “window current”, the classical Na_V_1.1 sodium current, does not have such characteristics. For this reason, the functioning of the Na_V_1.8 channels can be investigated by the Almers method, which is better known as the Almers limiting slope procedure [6,23]. The essence of this method, which can be successfully applied to study the behavior of the Na_V_1.8 channel activation gating system, is as follows. The voltage dependence of Na_V_1.8 channel conductance is rather steep, which indicates that the amino acid sequence of the channel molecule contains a voltage sensor detecting the E value. This region should bear an electric charge that could be displaced upon a depolarizing step change in the E value, consequently inducing a conformational transition of the channel gating system from the closed to the open state. It is known that the background intensive parameter thermodynamically coupled to the extensive parameter E is the charge displacement required to open a single channel, Z_eff_, which is expressed in elementary charge units. Although the real value of Z_eff_ is not known with certainty, it seems intuitively to be related to the steepness with which the sodium conductance depends on the membrane potential, and it can be evaluated using a very simple idea by introducing the L(E) function. This approach was described in detail earlier [6,24]. The L(E) function is plotted based on the formula below:

$$\underset{E\to -\infty }{\lim}L(E)=\underset{E\to -\infty }{\lim}\ln\frac{G_{Na}\left(E\right)}{G_{Na}^{max}–G_{Na}\left(E\right)}\underset{E\to -\infty }{\to }const\cdot \exp\left(\frac{Z_{eff}\cdot e_{0}\cdot E}{k\cdot T}\right),$$

where G_Na_(E) is the voltage dependence of the Na_V_1.8 channel chord conductivity, G^max^_Na_ is its maximum value, E is the membrane potential, k is the Boltzmann constant, and T is the absolute temperature. 

According to the Almers theory, Z_eff_ can be easily evaluated from the tangent of the slope of the asymptote passing through the very first points of the L(E) function due to the application of the Boltzmann distribution. The ratio G_Na_(E)/(G^max^_Na_ − G_Na_(E)) displays the voltage dependence of the distribution between the numbers of open (N_o_) and closed (N_c_) Na_V_1.8 channels. Indeed, G_Na_(E) is assumed to be proportional to N_o_, while G^max^_Na_ − G_Na_(E) is assumed to be proportional to N_c_. Macroscopic characteristics of the Na_V_1.8 channel current–voltage function can be thus used to evaluate the microscopic molecular parameter, Z_eff_, which controls the behavior of the Na_V_1.8 channel activation gating system and excitability of the nociceptive neuron membrane.

The patch-clamp method was implemented in the “whole-cell recording” configuration [25] using the hardware–software setup: the patch-clamp L/M-EPC 7 amplifier, digital–analog and analog–digital converters, and the computer. The accuracy of Z_eff_ evaluation strongly depends on the correctness of the patch-clamp method application. Both dynamic and stationary errors of the patch-clamp method are determined by the series resistance R_S_, which is calculated automatically during the experiment. Its value should be less than 3 MΏ because the stationary and kinetic parameters of the currents are otherwise obtained with large errors, as demonstrated by theoretical analysis of limitations of the patch-clamp method applicability [26].

### 2.3. Organotypic Tissue Culture Method

The Ac-KEKK-NH_2_ effect on neurite growth was studied on DRG explants from 10 to 12-day-old chicken embryos. Explants were placed in collagen-coated sterile Petri dishes and cultured in the medium composed of 45% Hank’s solution and 40% Eagle’s medium supplemented with insulin (0.5 U/mL), glucose (0.6%), L-glutamine (2 mM), gentamicin (100 U/mL), 10% fetal bovine serum, and Ac-KEKK-NH_2_ at the studied concentrations for three days in the CO_2_ incubator (Sanyo, Osaka, Japan) at 37 °C and 5% CO_2_. Control explants were cultured without Ac-KEKK-NH_2_. Explants were visualized with an Axio Observer Z1 microscope (Carl Zeiss, Oberkochen, Germany) and analyzed with ImageJ (National Institutes of Health, Bethesda, MD, USA) and ZEN_2012 (Carl Zeiss, Germany) software to evaluate the neurite growth using the area index (AI), which uses the ratio of the peripheral growth zone area to the central zone area. The average AI value in the control explants was taken as 100% [9]. Experiments were conducted using the equipment of the Confocal Microscopy Collective Use Center at the Pavlov Institute of Physiology, Russian Academy of Sciences.

### 2.4. Conformational Analysis

The TINKER 8.0 program package [27] with the MMFF94 force field [28] was used to carry out the conformational analysis of Ac-KEKK-NH_2_. The GB/SA approach [29] was chosen to take solvation effects into implicit account with the dielectric constant ε = 10.0 (the ε value models dielectric properties of the surrounding milieu at the moment of ligand–receptor binding of the peptide to the Na_V_1.8 channel) and ε = 80.4 (aqueous solution, physiologically adequate conditions). The algorithm of low-mode conformational search (LMOD) was implemented with about 100,000 single searches for each structure [30]. The amino groups of the lysine side chains were always positively charged. The carboxyl group of the glutamic acid side chain was negatively charged, but a possibility was also considered that it might be protonated and electrically neutral (uncharged) at ε = 10.0. 

The measure of distance between two amino groups is the distance between their nitrogen atoms. Statistical data processing was conducted using our custom C++ script over the entire ensemble of ~100,000 conformations and several subensembles, which contained all conformations with energies not exceeding a certain cutoff value relative to the global minimum. A total of 7 subensembles were created based on the cutoff values of 2, 3, 4, 4.5, 5, 6, and 7 kcal/mol. The methodology of data processing is described in more detail earlier [8,9].

### 2.5. Formalin Test

The formalin test is a traditional model of tonic inflammatory pain [31,32,33,34] that is widely used to assess the efficacy of novel analgesic medicinal substances [35,36,37,38] and to study the mechanisms through which tonic nociception is generated. The subcutaneous injection of formalin into the pad of a hind paw induces easily reproducible types of behavior caused by pain (flexing, shaking, and licking the paw); the formalin-induced response is organized into two phases. Flexing and shaking behaviors are organized at the spinal level, and licking behavior is at the supraspinal. The formalin test allows assessing acute short pain (first phase, duration of 5–10 min) and tonic prolonged pain (second phase, duration of 30–40 min). 

The study of the antinociceptive effect of Ac-KEKK-NH_2_ was carried out by the previously described protocol [34]. For the experiment, adult Wistar male rats (n = 16) were used with an average mass of 230 g. Experimental rats (n = 8) were exposed to Ac-KEKK-NH_2_ diluted in Hank’s solution (3.0 mg/kg, 1 mL, intraperitoneally); control rats (n = 8) received the same volume of Hank’s solution only (1 mL, intraperitoneally). Then, 5 min after the medicinal substance injection, each animal received a subcutaneous injection of formalin (2.5%, 50 μL) into the pad of the left hind paw, and then, the rat was immediately placed in an experimental chamber (25 × 25 × 25 cm) with transparent walls; the chamber was surrounded with mirrors. The registration of formalin-induced behavior indices (the number of flexes + shakes and licking duration) was performed for 60 min after the formalin injection using special software, which allows recording, quantifying, and analyzing the pain-related behavior.

### 2.6. Statistical Analysis 

The data were analyzed with STATISTICA 10.0 (StatSoft, Inc., Tulsa, OK, USA) using Student’s t-test and expressed as the mean value ± SEM. Statistical significance was set at *p* < 0.05.

## 3. Results

### 3.1. The Patch-Clamp Method

**Figure 1:**
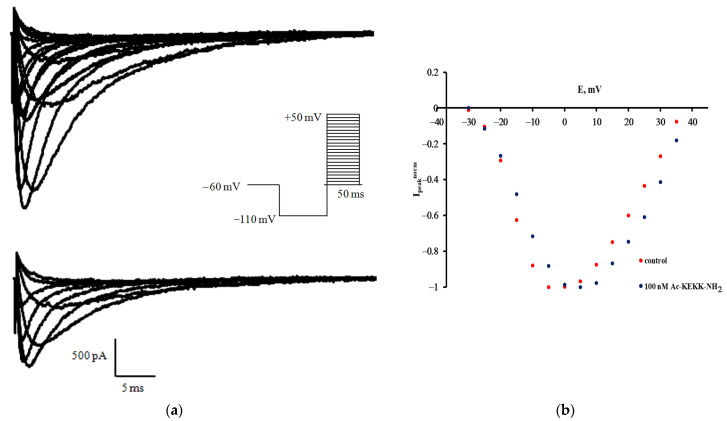
Effect of Ac-KEKK-NH_2_ on the Na_V_1.8 channels. (**a**) Families of slow sodium Na_V_1.8 currents in the control experiment (above) and after the application of Ac-KEKK-NH_2_ at 100 nM (below). (**b**) Normalized peak current–voltage characteristics of the Na_V_1.8 channels in the control experiment and after the application of Ac-KEKK-NH_2_ at 100 nM. The test potential was changed from –60 to 45 mV with a step of 5 mV. The holding potential of 300 ms duration was equal to –110 mV in all records. The leakage and capacitive currents were subtracted automatically.

**Figure 2:**
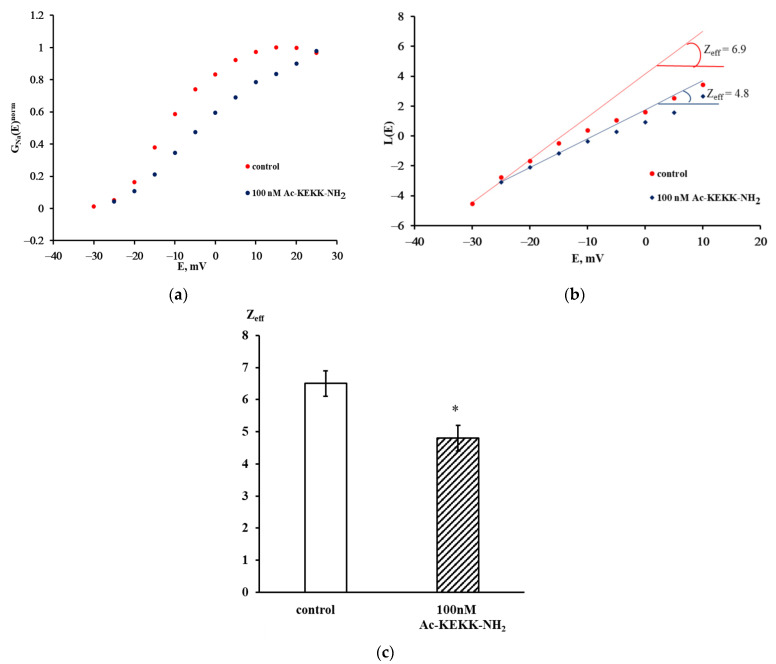
Effect of Ac-KEKK-NH_2_ on voltage sensitivity of the Na_V_1.8 channel activation gating system. (**a**) Normalized voltage dependence of Na_V_1.8 channel chord conductance G_Na_^norm^(E). (**b**) Evaluation of the effective charge transferred by the Na_V_1.8 channel activation gating system (Z_eff_) using the Almers logarithmic limiting voltage sensitivity L(E) function. (**c**) Values of Z_eff_ transferred by the Na_V_1.8 channel activation gating system in the control conditions and after the application of Ac-KEKK-NH_2_. Data are presented as mean ± SEM. Statistically significant difference between the control and experimental values is designated with the asterisk (*p* < 0.05).

The effects of Ac-KEKK-NH_2_ on the Na_V_1.8 channel voltage sensitivity were investigated by the patch-clamp method. In the course of the experiment, the families of Na_V_1.8 sodium currents arising in response to the protocol of voltage impulses were registered in the control conditions and after the application of Ac-KEKK-NH_2_ at 100 nM (Figure 1a). The decrease in the amplitude values of sodium currents observed after the action of Ac-KEKK-NH_2_ is due to the rundown effect inherent to the patch-clamp method. Normalized peak current–voltage characteristics of Na_V_1.8 currents are presented in Figure 1b. The target effect that makes it possible to establish a quantitative relationship between the change in voltage sensitivity of the Na_V_1.8 channel activation gating system and the application of an agent is manifested in a change of the steepness of the current–voltage function left branch. It can be visualized more clearly if the voltage dependence of the chord conductance G_Na_(E) is constructed as follows:

G_Na_(E) = I_max_(E)/(E − E_Na_),

where E_Na_ is the reversal potential for sodium ions, and I_max_(E) is the amplitude value of the sodium current generated in response to the step of depolarizing potential E. The G_Na_(E) function has the initial S-shaped branch, the slope of which describes the distinctive features of voltage sensitivity of the activation process (Figure 2a). The protocol of Z_eff_ evaluation from the Almers logarithmic limiting voltage sensitivity L(E) function is presented in Figure 2b. The tangents of the slopes of the asymptotes passing through the first three points of the L(E) function determine the limiting logarithmic sensitivity of the Na_V_1.8 channel to the transmembrane potential change. The asymptotes to the L(E) functions have different slopes in the control experiment and after the application of Ac-KEKK-NH_2_, which indicates a change in the Z_eff_ value. The Z_eff_ value decreased from 6.9 elementary charge units in the control experiment to 4.8 after the application of Ac-KEKK-NH_2_ (Figure 2b). 

The effective charge of the Na_V_1.8 channel activation gating system Z_eff_ was evaluated for each of 27 neurons before and after the Ac-KEKK-NH_2_ application using the Almers method. This method is based on measuring the chord conductance of an isolated neuron, which, in turn, is obtained from its current–voltage function. A passage to the limit is carried out further; i.e., the Almers logarithmic limiting voltage sensitivity L(E) function is constructed. It should be stressed that statistical data processing makes sense only from this step because the current–voltage functions of neurons can have different shifts along the voltage axis E. The position of the current–voltage function extremum depends on the series resistance R_S_, which is an individual characteristic of a neuron. However, this phenomenon does not affect the accuracy of Z_eff_ evaluation if R_S_ is maintained under 3 MΩ throughout the experiment. To add further, the R_S_ value might increase during the experiment. Considering this, only the Z_eff_ values are valid to be statistically processed [8,23].

The ensemble-averaged data displayed in Figure 2c indicate that the extracellular application of Ac-KEKK-NH_2_ at 100 nM to the nociceptive neuron membrane results in a significant decrease in the Z_eff_ value from 6.5 ± 0.4 (n = 27) in the control experiments to 4.8 ± 0.4 (n = 27) after the agent has been applied. The two means are significantly different (t = 3.01, *p* = 0.0041). A similar decrease in the Z_eff_ value from 6.4 ± 0.3 (n = 17) to 4.6 ± 0.3 (n = 23) has been observed earlier upon the application of Ac-RERR-NH_2_ at 100 nM [7]. 

### 3.2. Organotypic Tissue Culture Method

**Figure 3:**
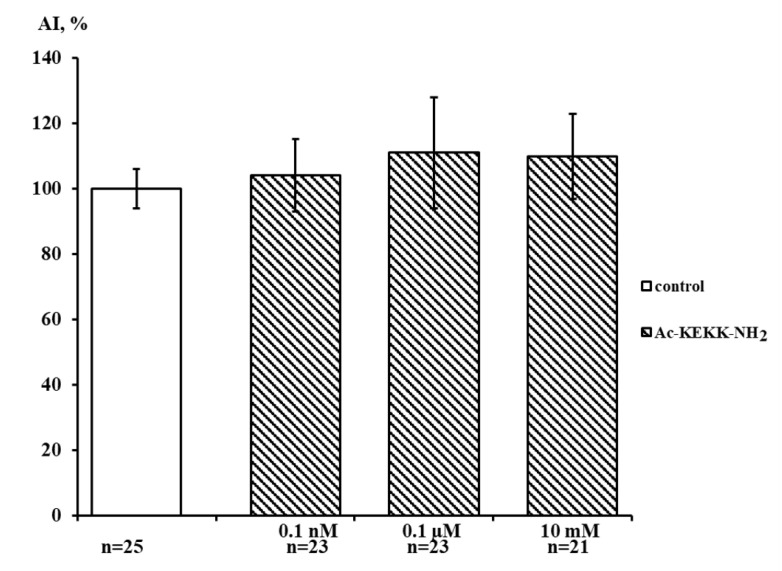
Effect of Ac-KEKK-NH_2_ on DRG neurite growth. The ordinate axis—area index (AI, %). Data are presented as mean ± SEM (not significant, *p* > 0.5).

Applied at the concentrations of 0.1 nM, 0.1 μM, and 10 mM, Ac-KEKK-NH_2_ did not exhibit any statistically significant effect on dorsal root ganglia neurite growth. The area index (AI) of experimental explants was close to the control value (Figure 3). The data obtained strongly suggest that the studied peptide does not trigger the Na,K-ATPase-coupled signal transduction pathways discovered by us earlier which modulate the Na_V_1.8 channel functioning in the nociceptive neuron membrane [6]. This signaling mechanism affects the cell genome and controls neurite growth, so its activation is expected to result in a change in the AI value as compared with the control. 

### 3.3. Conformational Analysis

**Figure 4:**
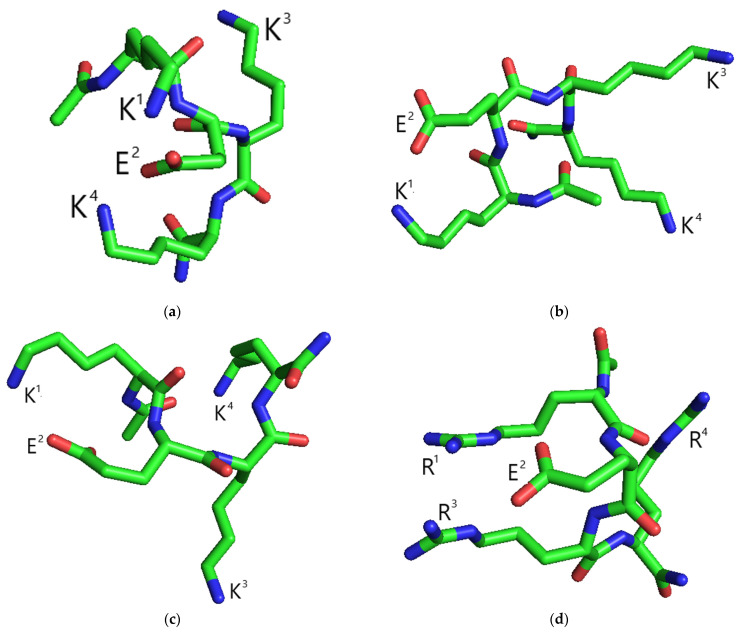
Lowest energy conformations of the peptides obtained by conformational analysis. (**a**) Ac-KEKK-NH_2_ ch, ε = 10; (**b**) Ac-KEKK-NH_2_ ch, ε = 80; (**c**) Ac-KEKK-NH_2_ unch, ε = 10; (**d**) Ac-RERR-NH_2_ ch, ε = 10. Carbon, green; oxygen, red; nitrogen, blue. Hydrogen atoms are not shown. Data for Ac-RERR-NH_2_ are taken from [9].

At ε = 80, which models physiologically adequate conditions, the carboxyl group of the glutamic acid side chain was charged negatively (this molecular form is further designated as Ac-KEKK-NH_2_ ch). At ε = 10, which models the protein environment at the moment of ligand–receptor binding of the attacking peptide with the Na_V_1.8 channel molecule, the carboxyl group was also considered uncharged because its protonation state was not a priori known in this case (Ac-KEKK-NH_2_ unch). The lowest energy conformations of the studied Ac-KEKK-NH_2_ molecular forms are shown in Figure 4. As follows from the figure, the spatial organization of the functional groups in the molecules can be very diverse, which illustrates that an adequately selected ensemble of conformations is required to numerically analyze the structural features of the peptides. The overall peptide structure is mainly determined by electrostatic repulsion between the amino groups. Both the ε value and protonation state of the glutamic acid carboxyl group have little effect on the values of distances between the lysine amino groups averaged over the entire ensemble of conformations (Table 1). Monitoring how these values are correlated with a gradual decrement of the energy cutoff helps estimate the optimal distances between the amino groups for effective ligand–receptor binding of the attacking peptide. Logically, many low-energy conformations are expected to be stabilized by intramolecular ionic bond(s) between the glutamic acid carboxyl group and lysine amino groups, which brings the above functional groups more closely together and creates a significant energy barrier for their participation in ligand–receptor ionic bonds with the Na_V_1.8 channel. Indeed, if the glutamic acid carboxyl group bears a negative charge, a drastic decrease in the K^1^–K^3^ distances is observed when the energy cutoff goes down from 4.5 to 4 kcal/mol. This supports our prior suggestion that a subensemble required to realistically estimate the distances between the amino groups in the low-energy conformational space should include at least 1% of the total count of conformations, around 1000 [8,9]. The K^1^–K^3^ (11–12 Å) and K^3^-K^4^ (~11 Å) distances remain fairly constant if the energy cutoff value is higher than 5 kcal/mol. The K^1^–K^4^ distance value also decreases to ~11 Å in the low-energy subensembles, which is ~2 Å less than the corresponding value obtained by averaging over the entire ensemble (Table 1). Thus, positively charged lysine amino groups in the Ac-KEKK-NH_2_ molecule form an almost equilateral triangle with a side length of 11–12 Å.

### 3.4. Formalin Test

**Figure 5:**
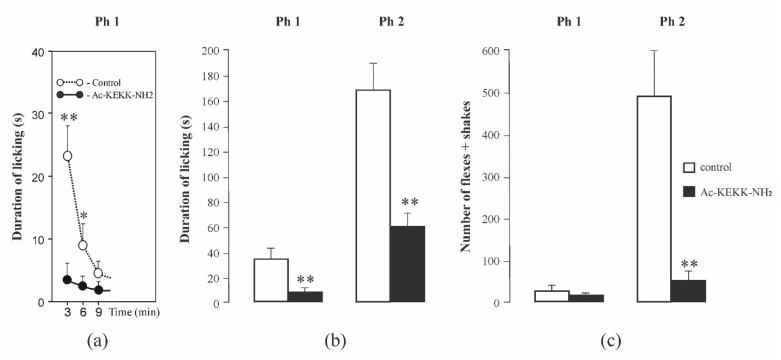
Effect of Ac-KEKK-NH_2_ on licking duration and the number of flexes + shakes in the formalin test. (**a**) The dynamics of licking duration in the acute phase (Ph 1) after the subcutaneous injection of formalin, abscissa, the time after formalin injection; (**b**) licking duration in the acute (Ph1) and tonic (Ph2) phases; (**c**) the number of flexes + shakes in the acute (Ph1) and tonic (Ph2) phases. Data are presented as mean ± SEM. Statistically significant differences between the control and experimental values are designated with asterisks (* *p* < 0.05, ** *p* < 0.01).

The intraperitoneal administration of the lysine-containing peptide Ac-KEKK-NH_2_ (3.0 mg/kg) to the experimental rats five minutes prior to the subcutaneous formalin injection caused an immediate decrease in licking duration in the first six minutes of formalin-induced pain. The dynamics of licking duration in the first acute phase (Ph 1) as compared to the control is presented in Figure 5a. Ac-KEKK-NH_2_ reduced the duration of licking also in the second prolonged tonic phase (Ph 2); i.e., it relieved pain. The mean values of licking duration in the experimental and control rats in the first acute (6.4 ± 3.9 vs. 34.2 ± 8.1, t = 3.09, *p* = 0.008) and second tonic (60.9 ± 10.4 vs. 176.9 ± 21.5, t = 4.54, *p* < 0.001) phases are presented in Figure 5b. No significant difference between the experimental and control animals was found in the number of flexes + shakes during the acute phase (17.0 ± 7.0 vs. 31.0 ± 8.4, t = 1.27, *p* = 0.2372). However, during the tonic phase, a significant decrease in the number of flexes + shakes was observed in the experimental animals (55.6 ± 18.7 vs. 490.4 ± 109.9, t = 3.90, *p* = 0.0016) (Figure 5c). Thus, the analgesic effect of Ac-KEKK-NH_2_ was demonstrated in formalin-induced behavior organized at both the spinal and supraspinal levels of the central nervous system.

## 4. Discussion

The entire methodology of our patch-clamp research is based on the postulate that the opening of the activation gating system occurs during the transition of the Na_V_1.8 channel from the single closed state to the open state, of which there may be several, and this transition itself is unique. In this case, the analysis of experimentally obtained data can be independent of the activation gating system model [23]. The above postulate holds for the beginning of the activation process; therefore, the logarithmic function L(E) can be realistically fitted by a linear asymptote only in its very initial part (Figure 2b). The further nonlinear behavior of the L(E) function reflects the involvement of other open states in the Na_V_1.8 channel activation process. An essentially important consequence of this postulate is that the Boltzmann distribution can be applied to describe the voltage dependence of the distribution between open and closed channels in the ensemble, which has a physical meaning. Because our methodological approach ensures that only the Na_V_1.8 channels are included in the ensemble, the effective charge transferred by the activation gating system of a single Na_V_1.8 channel can be evaluated.

The nonlinear displacement sodium currents recordings method [39] cannot provide a reliable answer regarding whether the Na_V_1.8 channel activation gating system in the nociceptive neuron is directly involved in ligand–receptor binding of the lysine- and arginine-containing short peptides, specifically modulating the functioning of these channels [8,9]. The gating currents registered by the Armstrong–Bezanilla method cannot be unambiguously attributed to the responses of the Na_V_1.8 channels. Other charged membrane proteins also can become displaced within the neuron membrane, which makes it hardly possible to evaluate the effective charge transferred by the Na_V_1.8 channel activation gating system with sufficient precision. This difficulty can be overcome only by applying the Almers method, which is appropriate when the gating system of the ion channel is characterized by the single transition from the closed to the open state at negative values of the membrane potential E. The behavior of the Na_V_1.8 channel activation gating system ideally fits the above criterion. Moreover, as opposed to the fast classical Na_V_1.1 channels, the steady-state Na_V_1.8 activation and inactivation gating processes are separated along the E axis, which makes the experimental construction of the Almers limiting conductivity function L(E) the most efficient methodology to evaluate Z_eff_ [6]. Such an approach allows us to predict that the agents decreasing the Na_V_1.8 channel functional activity should exhibit an antinociceptive effect at the organismal level. The presented results of behavioral tests (Figure 5) support this hypothesis.

The implementation of our methodological approach to study the effects of arginine-containing short peptides has made it possible to establish that the positively charged guanidinium functional groups are responsible for their ligand–receptor binding to the Na_V_1.8 channels encoding the nociceptive information [8,9]. Average distances between the guanidinium groups have been demonstrated to fall within a certain range with the lower threshold of ~9 Å to provide ligand–receptor complementarity. Similar logic should apply to the investigation of the ligand–receptor binding mechanism of lysine-containing short peptides. However, there is a substantial difference between the amino and the guanidinium groups with regard to the distribution of positive electric charge over the nitrogen atoms present in the corresponding moiety. The lysine side-chain amino group can be considered as a point charge because its positive charge is localized on the active nitrogen atom, which is the only heavy atom in this functional group. Therefore, the distances between the amino groups describe the distances between atoms that directly participate in ligand–receptor ionic bonds. The positive charge of the guanidinium group is delocalized over three potentially active nitrogen atoms centered around the carbon atom. Because it is not known which nitrogen atom(s) of a guanidinium group is involved in intermolecular ionic bonds with the Na_V_1.8 channel molecule, a 3 × 3 matrix would be required to correctly represent all possible pairwise distances between nitrogen atoms in two guanidinium groups. This would make it hardly possible to build a scale to compare the structural parameters of different arginine-containing peptides and correlate their values with the Z_eff_ values obtained by the patch-clamp method. That is why we had to choose the distance between the inactive carbon atoms positioned close to the geometry centers of the guanidinium groups as the model measure of distance between these functional groups. As a consequence, it is difficult to draw direct conclusions from the single example studied herein about how average distance values are expected to change when arginines are substituted for lysines in a peptide molecule, and further investigation of the lysine-containing short peptides is required to correlate their structural parameters with their physiological effects. In the present case, average distance values between nitrogen-containing side-chain functional groups in the Ac-KEKK-NH_2_ molecule are ~2–3 Å larger than those in the Ac-RERR-NH_2_ molecule (see Table 1).

The current work reports the pioneer study of a lysine-containing short peptide. It could not be a priori expected that the lysine-for-arginine substitution would keep the peptide molecule active because the lysine amino group is structurally different from the arginine guanidinium group. It has not been obvious whether the distances between the amino groups in the Ac-KEKK-NH_2_ molecule would correlate with the previously calculated distances between the guanidinium groups in the molecules of arginine-containing short peptides. Finally, another amino acid (lysine) has been established as a functional building block that might facilitate the further design and synthesis of novel effective lysine- and arginine-containing analgesic peptides, because fine-tuning of the steric and electrostatic ligand–receptor complementarity at the atomic level is required to effectively modulate the Na_V_1.8 channel activation gating system.

One of the major conclusions of the present work is that the ligand–receptor interaction of lysine- and arginine-containing short peptides with the Na_V_1.8 channel molecule is controlled by intermolecular bonds of a primarily electrostatic nature, while hydrogen bonds are not expected to play a prominent role in the process. The guanidinium group of arginine, in addition to being positively charged, has a large potential for hydrogen bond formation due to the relatively large molecular volume of the moiety, whereas this potential can hardly be realized in the case of the lysine amino group. Both the above peptides, applied at the same concentration of 100 nM, have been shown to effectively decrease the Z_eff_ value of the Na_V_1.8 channel activation gating system, indicating that the chemical structure of positively charged side-chain functional groups present in the attacking molecules should not be so important for their effective ligand–receptor binding with the Na_V_1.8 channel. A much more important factor is the presence of a positive electric charge at the end of a long aliphatic side chain, which facilitates the steric and electrostatic accommodation of the peptides in their binding site. Therefore, it is the glutamic acid or the aspartic acid side-chain carboxylate anions of the Na_V_1.8 channel molecule that participate in ionic bonds with the attacking cationic peptide. Given that the distance range between the positive charges in the active peptide molecules is determined by conformational analysis, it might facilitate the search for the peptide-binding site in the Na_V_1.8 channel. 

The absence of any significant effect of Ac-KEKK-NH_2_ on neurite growth observed in the organotypic tissue culture experiments indicates that this lysine-containing short peptide does not activate membrane metabotropic receptors which trigger downstream signaling cascades controlling the neurite growth. This allows us to predict that the substance specifically binds to the Na_V_1.8 channel. 

The delicate molecular mechanisms of modulation of the Na_V_1.8 channel functioning by the lysine- and arginine-containing short peptides discovered by us are crucially important from the practical viewpoint. Our data show that the investigated short peptide is effective at the supraspinal level, which means that the Ac-KEKK-NH_2_ molecule probably crosses the blood–brain barrier due to its endogenous nature. More likely, in our opinion, it is another physiological mechanism of action of the lysine-containing short peptide at the supraspinal level. We have shown earlier that the effect of comenic acid is exhibited not only due to direct interaction with supraspinal neurons but also due to a change in the impulse firing of peripheral neurons where the Na_V_1.8 channels are functioning [40]. Our previous data also show that modulation of the Na_V_1.8 channel activation gating system by Anoceptin^®^ results in a strong effect on intracranial self-stimulation of the lateral hypothalamus [40].

The antinociceptive effect of Ac-KEKK-NH_2_ has been demonstrated in vivo in the behavioral tests at the organismal level, which makes this short peptide a very promising candidate for the role of a novel effective and safe analgesic medicinal substance, and this is another major conclusion of the current study. 

Twenty years ago, using the Almers method, we discovered a very strong analgesic effect of the endogenous arginine-rich antibiotic defensin NP-1 with the total charge of +9 that contains ten arginine residues out of 33 amino acids [41]. A part of its amino acid sequence, RERR, has been shown to modulate the Nav1.8 channel functional activity [9]. Hence, we succeeded in separating the analgesic effect of the defensin from its antibiotic activity. Moreover, we have predicted that lysine-containing short peptides could exhibit an antinociceptive effect due to activation of the same molecular mechanism. This is what we have demonstrated in the present work. Short peptides rarely exhibit toxic side effects [42,43,44]. It can be suggested with a high probability that the active peptide concentration chosen herein (100 nM) is safe due to the endogenous nature of the investigated short peptide.

The application of four very modern techniques within the framework of our unique methodological approach has made it possible to demonstrate that the analgesic effect should be exhibited by a cationic peptide which meets the following two structural criteria. The peptide should be able to participate in intermolecular ligand–receptor ionic bonds, and the distances between its positively charged functional groups should fall in the range of 9–12 Å. The indicated range has been identified as a result of decade-long investigations of the effects and structure of arginine-containing short peptides summarized in our recent publications [8,9]. We have designed, synthesized, and studied 10 short peptides. This allowed us to show that the Na_V_1.8 channel activation gating system is modulated only if the distance between the cationic functional groups exceeds 9 Å. Therefore, we have formulated a protocol to predict other peptide structures that could claim the role of an analgesic substance, which has been demonstrated in the current work. The further investigation of lysine-containing short peptides with different spacing between the amino groups carrying the point positive charge could help identify more precisely the upper threshold of the distance range between the cationic functional groups responsible for ligand–receptor interaction of the attacking peptides with the Na_V_1.8 channel molecule. 

## Figures and Tables

**Table 1:** Average distances between the lysine amino groups in the studied molecular forms of the Ac-KEKK-NH_2_ molecule.

Cutoff, kcal/mol	Ac-KEKK-NH2 ch		Ac-KEKK-NH2 ch		Ac-KEKK-NH2 unch		Ac-RERR-NH2 ch	
	ε = 10		ε = 80		ε = 10		ε = 10	
	Nconf	Distances, Å	Nconf	Distances, Å	Nconf	Distances, Å	Nconf	Distances, Å
None	102,834	K^1^–K^3^ 11.8 ± 2.9 K^1^–K^4^ 12.8 ± 3.7 K^3^–K^4^ 10.7 ± 2.1	102,656	K^1^–K^3^ 11.8 ± 2.9 K^1^–K^4^ 12.8 ± 3.7 K^3^–K^4^ 10.8 ± 2.1	102,348	K^1^–K^3^ 11.9 ± 2.9 K^1^–K^4^ 12.9 ± 3.6 K^3^–K^4^ 10.6 ± 2.2	101,546	R^1^–R^3^ 9.8 ± 3.4 R^1^–R^4^ 9.2 ± 3.7 R^3^–R^4^ 9.4 ± 2.7
7	3778	K^1^–K^3^ 12.0 ± 2.8 K^1^–K^4^ 11.3 ± 3.4 K^3^–K^4^ 11.0 ± 1.7	4109	K^1^–K^3^ 11.9 ± 2.7 K^1^–K^4^ 11.3 ± 3.5 K^3^–K^4^ 11.0 ± 1.7	7443	K^1^–K^3^ 12.1 ± 2.8 K^1^–K^4^ 12.1 ± 3.9 K^3^–K^4^ 11.0 ± 1.8	2354	R^1^–R^3^ 9.7 ± 3.1 R^1^–R^4^ 8.0 ± 2.8 R^3^–R^4^ 9.1 ± 2.4
6	1551	K^1^–K^3^ 11.9 ± 2.7 K^1^–K^4^ 11.1 ± 3.3 K^3^–K^4^ 10.9 ± 1.7	1803	K^1^–K^3^ 11.6 ± 2.7 K^1^–K^4^ 11.2 ± 3.4 K^3^–K^4^ 11.0 ± 1.7	3742	K^1^–K^3^ 12.2 ± 2.8 K^1^–K^4^ 11.8 ± 3.9 K^3^–K^4^ 11.0 ± 1.8	1023	R^1^–R^3^ 9.7 ± 3.1 R^1^–R^4^ 8.2 ± 2.7 R^3^–R^4^ 9.2 ± 2.3
5	579	K^1^–K^3^ 11.7 ± 2.7 K^1^–K^4^ 11.0 ± 3.3 K^3^–K^4^ 10.9 ± 1.7	711	K^1^–K^3^ 11.3 ± 2.7 K^1^–K^4^ 11.0 ± 3.3 K^3^–K^4^ 11.1 ± 1.7	1506	K^1^–K^3^ 12.3 ± 2.8 K^1^–K^4^ 11.3 ± 3.8 K^3^–K^4^ 10.9 ± 1.7	408	R^1^–R^3^ 9.3 ± 3.2 R^1^–R^4^ 8.2 ± 2.4 R^3^–R^4^ 9.3 ± 2.3
4.5	351	K^1^–K^3^ 11.6 ± 2.7 K^1^–K^4^ 10.8 ± 3.3 K^3^–K^4^ 10.8 ± 1.8	423	K^1^–K^3^ 11.3 ± 2.8 K^1^–K^4^ 10.9 ± 3.3 K^3^–K^4^ 11.0 ± 1.8	897	K^1^–K^3^ 12.4 ± 2.8 K^1^–K^4^ 11.1 ± 3.7 K^3^–K^4^ 10.8 ± 1.8	258	R^1^–R^3^ 9.1 ± 3.3 R^1^–R^4^ 8.1 ± 2.4 R^3^–R^4^ 9.4 ± 2.2
4	186	K^1^–K^3^ 10.8 ± 2.7 K^1^–K^4^ 10.9 ± 3.3 K^3^–K^4^ 10.8 ± 1.8	234	K^1^–K^3^ 10.8 ± 2.8 K^1^–K^4^ 10.7 ± 3.2 K^3^–K^4^ 10.9 ± 1.8	490	K^1^–K^3^ 12.5 ± 2.8 K^1^–K^4^ 10.9 ± 3.6 K^3^–K^4^ 10.8 ± 1.7	157	R^1^–R^3^ 8.6 ± 3.2 R^1^–R^4^ 8.2 ± 2.4 R^3^–R^4^ 9.5 ± 2.2
3	55	K^1^–K^3^ 10.4 ± 2.7 K^1^–K^4^ 10.6 ± 3.2 K^3^–K^4^ 11.0 ± 1.5	71	K^1^–K^3^ 10.1 ± 2.8 K^1^–K^4^ 10.8 ± 3.1 K^3^–K^4^ 11.1 ± 1.7	108	K^1^–K^3^ 12.6 ± 2.5 K^1^–K^4^ 11.1 ± 3.3 K^3^–K^4^ 10.9 ± 1.5	55	R^1^–R^3^ 7.6 ± 2.9 R^1^–R^4^ 8.7 ± 2.1 R^3^–R^4^ 9.9 ± 2.0
2	18	K^1^–K^3^ 9.8 ± 2.9 K^1^–K^4^ 11.1 ± 3.0 K^3^–K^4^ 10.9 ± 1.4	22	K^1^–K^3^ 9.9 ± 3.0 K^1^–K^4^ 11.0 ± 3.1 K^3^–K^4^ 10.8 ± 1.6	30	K^1^–K^3^ 13.3 ± 2.2 K^1^–K^4^ 11.1 ± 3.0 K^3^–K^4^ 10.9 ± 1.4	21	R^1^–R^3^ 6.3 ± 2.3 R^1^–R^4^ 8.3 ± 1.6 R^3^–R^4^ 9.8 ± 1.5

The subscripts “ch” and “unch” designate the protonation state of the glutamic acid side chain carboxyl group; it is either negatively charged and deprotonated or it is uncharged and protonated, respectively. Distances between the guanidinium groups in the molecule of Ac-RERR-NH_2_, the arginine-containing analog of Ac-KEKK-NH_2_, are taken from [9].

## Data Availability

Not applicable.

## Supplementary Materials

The following supporting information can be downloaded at: https://www.mdpi.com/article/10.3390/life13091800/s1.
