# Supplementary material for: Analgesic Effect of the Lysine-Containing Short Peptide Is Due to Modulation of the NaV1.8 Channel Activation Gating System
Source: Life (Basel). 2023 Aug 24;13(9):1800. doi: 10.3390/life13091800 (PMC10533133; doi:10.3390/life13091800)
Supplement: Supplementary file 1 [file life-13-01800-s001.zip › life-2522648-supplementary.pdf]

Structure: Ac-Lys-Glu-Lys-Lys-NH_2

Molecular mass: per free peptide 572.70 Da, monoisotopic mass 572.36 Da

Mass spectrometry (MS) data: $(M+H)^+$ 573.36 Da

High-performance liquid chromatography (HPLC) data: the content of the basic substance by optical density (λ 220 nm) >95%; analytical column Waters DeltaPac C18, 5μ, 100 A, 3.9×150 mm, with gradient 1–50% MeCN in 0.1% TFA

Manufacturer: LLC "Verta" 197110 Saint Petersburg, Levashovsky prospect, 12A

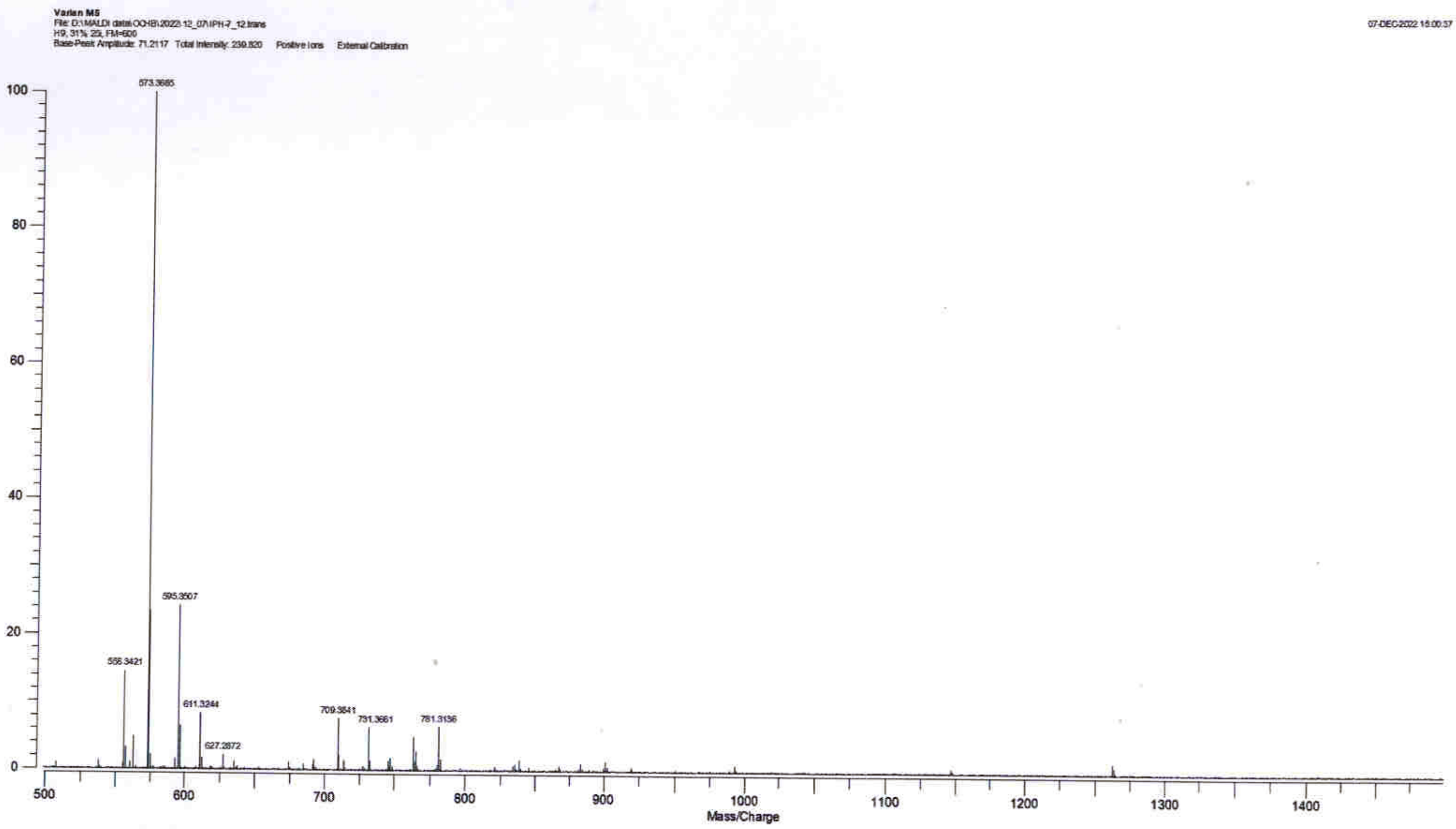

IPH-7, m.w. = 572.70, m.i. = 572.36

Analyzed on: Fri Dec 09 2022 13:38:31

Control Method Notes:
Column: Waters DeltaPak C18 100A 3.9x150mm 5u Mobile Phase:
0.1% TFA in water / MeCN - Flow Rate: 1ml / min - Detector: UV 220nm

Operations List Notes:
IPH 7

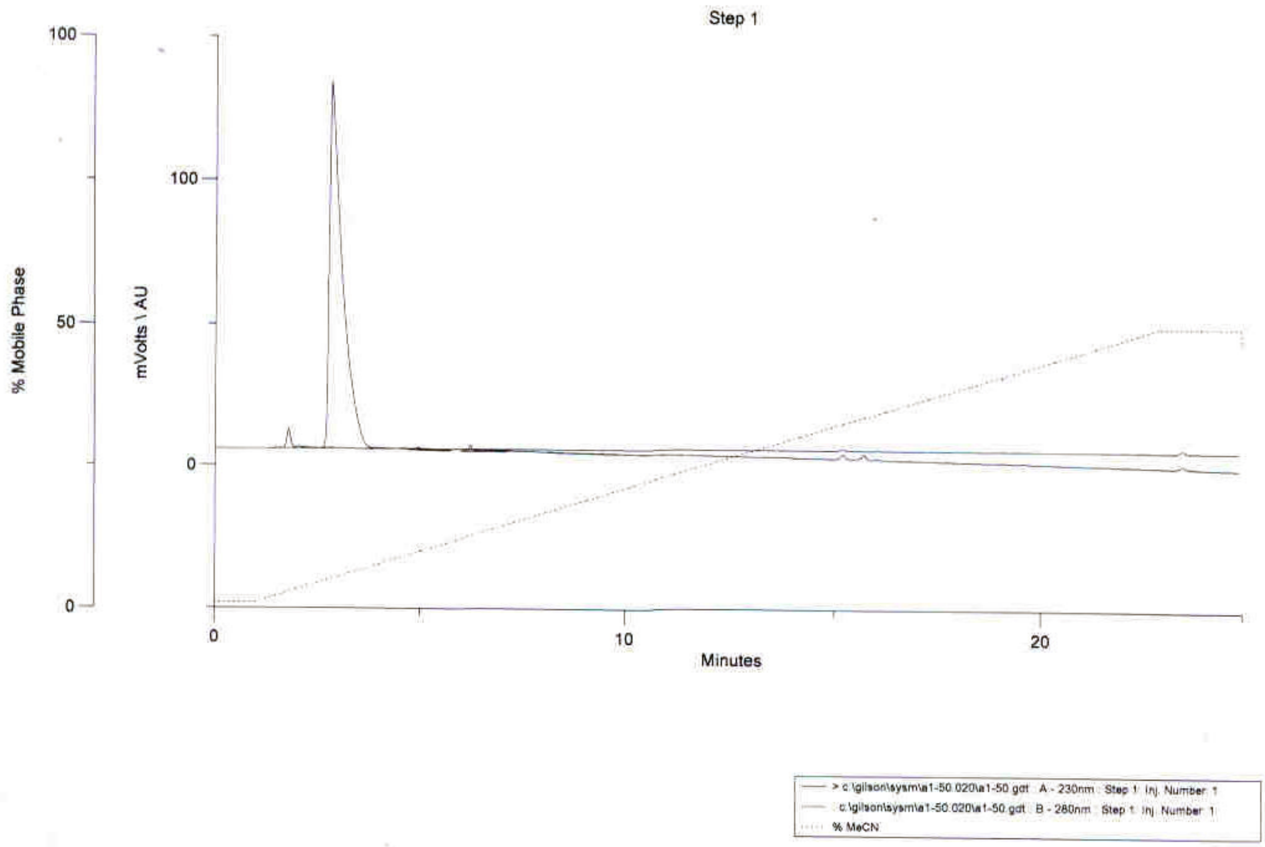

	R. Time	Area	Area %			
1	2.80	4611904	95.844			
2	4.95	64586	1.342			
3	6.23	69497	1.444			
4	8.22	32925	0.684			
5	9.45	32991	0.686			
